# Plant development influences dynamic shifts in the root compartment microbiomes of wild and domesticated finger millet cultivars

**DOI:** 10.1186/s12866-025-03976-8

**Published:** 2025-04-30

**Authors:** Fantaye Ayele Dadi, Saraladevi Muthusamy, Samrat Ghosh, Diriba Muleta, Kassahun Tesfaye, Fassil Assefa, Jie Xu, Farideh Ghadamgahi, Rodomiro Ortiz, Ramesh Raju Vetukuri

**Affiliations:** 1 Department of Plant Breeding, Swedish University of Agricultural Sciences, Alnarp, SE-234 22 Sweden; 2 Institute of Biotechnology, Addis Ababa University, Arat Kilo Campus, P. O. Box 1176, Addis Ababa, Ethiopia; 3 Institute of Biotechnology and DMCMB, Addis Ababa University, P. O. Box 32853 (Private Box), Arat Kilo Campus, Addis Ababa, Ethiopia; 4 Department of Microbial, Cellular and Molecular Biology, Addis Ababa University, Arat Kilo Campus, P. O. Box 1176, Addis Ababa, Ethiopia; 5 Department of Clinical Sciences, Lund University, Malmö, SE-202 13 Sweden

**Keywords:** Microbiome, Finger millet, Rhizosphere, Endosphere, Seedling stage, Flowering stage, Domestication, Amplicon sequencing, Plant microbiome, Plant microbiology

## Abstract

**Background:**

Plant-microbe interactions in the rhizosphere and endosphere are crucial for maintaining plant health and ecosystem dynamics. These interactions are shaped by several factors, including the plant’s developmental stage, domestication, and specific root compartments. Different plant cultivars influence unique microbial communities by secreting root exudates that either support beneficial symbionts or inhibit pathogens. This study examined the microbial community structures in the endosphere and rhizosphere of wild-type finger millet and five domesticated cultivars at two developmental stages.

**Results:**

Our results revealed that the plant developmental stage, root compartment, and domestication significantly influence the root-associated microbiomes. Interestingly, only about 8% of the core microbiota was consistently shared between the soil and plants, indicating that 92% shifted dynamically depending on plant type and developmental stage. *Pseudomonadota*, *Actinomycedota*, and *Bacteroidota* were the dominant bacterial phyla, while *Ascomycota* and *Basidiomycota* were the primary fungal phyla across all samples, displaying distinct abundance patterns. Notably, an increase in *Actinomycedota* in the endosphere correlated with a reduction in *Pseudomonadota*. The most significant shifts in microbial community composition occurred in the rhizosphere during the flowering stage, primarily driven by the genus *Pseudomonas*. These findings demonstrate that plant developmental stages and domestication influence the recruitment of specific microbial taxa to meet the plant’s needs, particularly in various root compartments. This selective recruitment highlights the active role of plants in shaping their microbiomes, providing insights into the potential for manipulating these communities to enhance crop productivity sustainably.

**Conclusion:**

Our results indicate that both the host developmental stage and domestication significantly influence the assembly and structure of the plant microbiome. Plant root compartments can selectively recruit specific taxa from associated core microbial communities to meet their needs, depending on the plant’s developmental stage and the particular root compartment involved. These findings demonstrate that the deterministic selection pressures exerted by plants during their growth and development greatly affect their microbial communities. This has important implications for developing sustainable farming practices, reducing reliance on chemical fertilizers and pesticides, and enhancing future crop productivity.

**Supplementary Information:**

The online version contains supplementary material available at 10.1186/s12866-025-03976-8.

## Background

Finger millet (*Eleusine coracana*) is an essential cereal crop renowned for its resilience and nutritional value, particularly in semiarid and tropical regions. It is the sixth most important cereal crop globally, with an annual production of around 4.5 million metric tons. It has been cultivated since the establishment of the earliest indigenous African communities [1]. Primarily cultivated in Africa and Asia, finger millet is a staple food and feed source, especially in developing nations [2–4]. Millet grains are rich in essential macronutrients, minerals, and polyphenols, and their nutritional value exceeds that of conventional cereals such as rice and wheat [2, 4, 5]. Moreover, the resilience of finger millet in harsh environments with poor soil fertility and elevated salinity allows it to flourish in areas where conventional agriculture is impractical.

Finger millet plants’ adaptability to various environmental conditions is partially mediated through their interactions with diverse soil microorganisms residing in different plant compartments, such as roots, stems, and seeds. Each compartment’s unique microenvironment influences these interactions [6]. The plant microbiome, particularly the root-associated microbiome, plays a major role in plant health, growth, and stress resilience. The composition and diversity of microbial communities vary notably between different compartments, such as the rhizosphere and endosphere, which are the primary microbial habitats in the root system [7].

The rhizosphere refers to the root surface and adjacent soil particles directly influenced by root secretions, while the endosphere relates to the roots’ internal root tissue [8]. These compartments differ in their environmental conditions, such as the availability of carbon sources, the content of host plant-derived compounds, and exposure to various climatic factors, leading to distinct microbial compositions [9, 10]. Consequently, the composition of the rhizosphere microbiota differs significantly from that of the endosphere. Generally, the rhizosphere harbors a more diverse microbiota than the endosphere [11].

Plant-associated microbiomes substantially influence plants’ phenotypic adaptability and the functionality of their root systems. The composition of these microbial communities may depend on several factors, including soil type, environmental factors, cultivar variation, plant development, and domestication. Moreover, the process of rhizodeposition, which involves the release of root exudates containing sugars, amino acids, and secondary metabolites, plays a vital role in selecting and modulating microbial communities in the rhizosphere. It allows specific microbial taxa to colonize the root surface, influencing the overall microbial community structure and function.

The microbiome composition within the plant compartment is highly variable and influenced by multiple factors [12]. Several recent studies on the microbiomes of the root compartments (i.e., the endosphere and rhizosphere) and soil in various crops have provided valuable insights into the structure and function of the microbial communities present in and around the roots [13–16]. The rhizosphere microbiome is dominated mainly by bacterial phyla such as *Proteobacteria* (new name: *Pseudomonadota*), *Acidobacteria (Acidobacteriota*), *Verrucomicrobia* (*Verrucomicrobiota*), *Bacteroidetes* (*Bacteroidota*), *Planctomycetes* (Planctomycetota), and *Actinobacteria* (*Actinomycetota*) [17]. In contrast, the endosphere is enriched with *Actinobacteria* (*Actinomycetota*) and fungal phyla *Ascomycota* and *Basidiomycota* [18]. Moreover, the specific microbial communities within these compartments can shift depending on plant developmental changes, genetic and cultivar-specific factors, and environmental conditions such as soil type and exposure to biotic and abiotic stresses [10, 15, 16, 19–21].

Plant domestication profoundly influences the composition and diversity of the microbiomes associated with different root compartments, including the rhizosphere and endosphere [22]. During domestication, selective breeding for traits such as improved yield, disease resistance and stress tolerance has remarkably influenced the associated microbial communities. The domesticated plant varieties often exhibit reduced microbial diversity than their wild type due to the diverse range of root exudates and modified immune signaling that selectively recruit or exclude specific microbial taxa [20, 23]. The variation in the root exudates is a major factor that drives the microbiome changes in domesticated plants. It may exude different types and quantities of sugars, amino acids, organic acids, and secondary metabolites, which serve as carbon sources and signaling molecules for soil microorganisms [19]. Such changes can reduce the diversity of the rhizosphere microbiome but may selectively enrich beneficial microbes, such as those involved in nutrient acquisition or pathogen suppression [12, 24].

Moreover, Domesticated plants often impose stronger selective pressures on their rhizosphere microbiome, favoring microbial taxa that can utilize specific exudates or withstand plant immune responses [25]. In the endosphere, shifts in microbial communities may be driven by changes in root architecture, cell wall composition, or immune-related genes [12, 20]. The selection and/or enrichment of specific microbes is a profound outcome of domestication. Microbes that improve nutrient uptake, growth, or disease resistance tend to be enriched in domesticated varieties, reflecting co-evolution between plant genotypes and their associated microbiomes [23, 26, 27]. Domesticated plants like maize and wheat effectively recruit beneficial rhizobacteria, optimizing nutrient acquisition and pathogen defense [23, 27]. However, the impacts of domestication on microbiome assembly are highly variable and vary from one crop to another, as reported in other studies [10, 15, 23, 28–30].

Hence, the microbiota composition and its relationship with the host plant are complex, dynamic, and plant-specific; in this study, we aimed to determine how the host plant’s developmental stage and genetic background shape the root-associated microbiota in finger millet. To this end, we screened the microbiota of wild-type finger millet and five domesticated cultivars. We focused on the bacteria and mycobiota and their changes during the seedling and flowering Plant developmental stages.

## Results

### Microbial composition of the endosphere and rhizosphere during the seedling and flowering stages

**Fig. 1:**
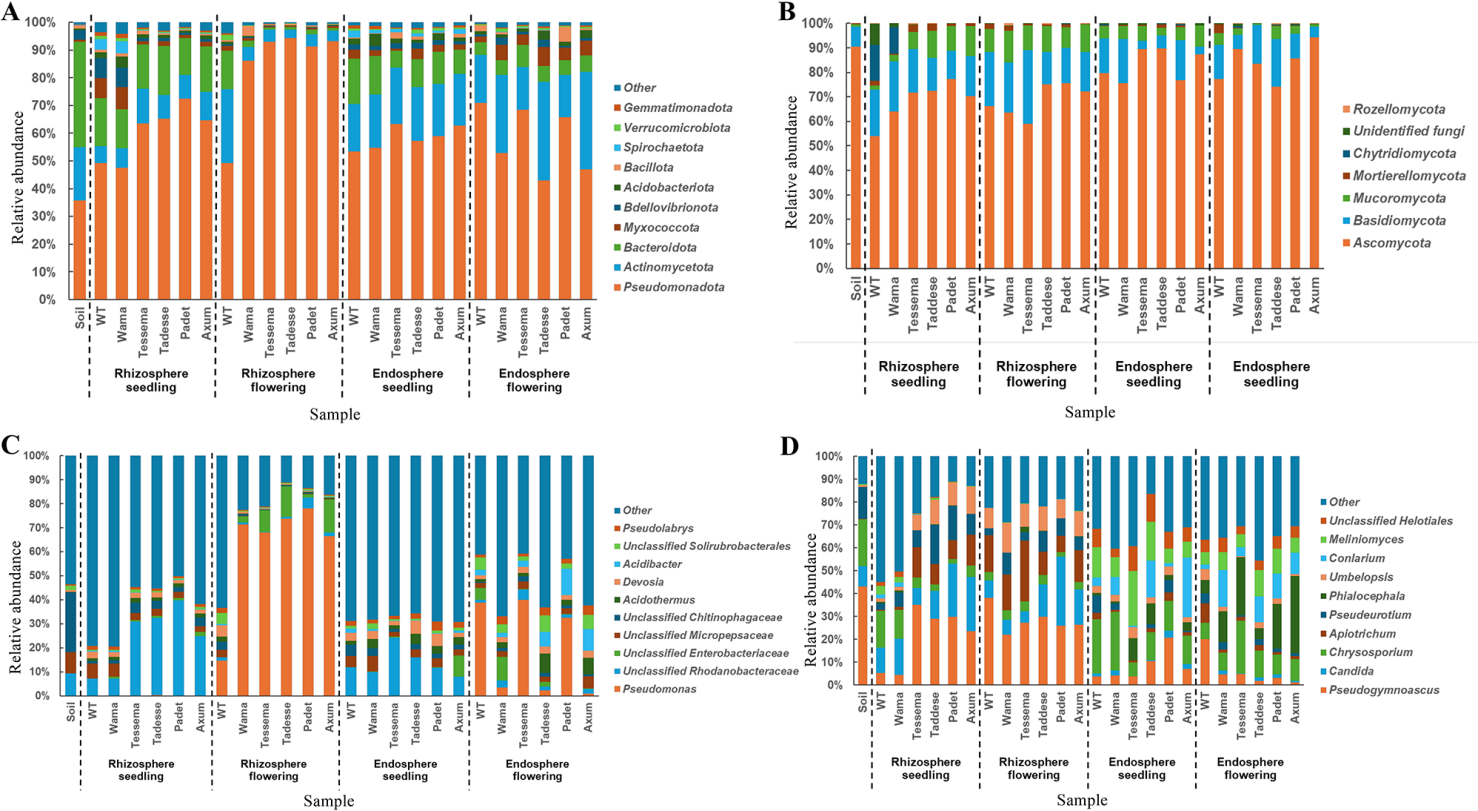
Comparative Analysis of Bacteriota and Mycobiota Across Plant Compartments. This figure shows the top ten bacterial phyla (**A**), all detected fungal phyla (**B**), the top ten bacterial genera (**C**), and the top ten fungal genera (**D**) identified in the endosphere and rhizosphere of plants during the seedling and flowering stages. The samples compared include control (soil without plantation), wild type (Africana), and five different cultivars (Wama, Tessema, Tadesse, Padet and Axum). Relative abundances > 0 are shown in the heat maps of (**C**) and (**D**). The X-axis represents the sample’s names, and the Y-axis indicates the relative abundance values of the corresponding phylum and genus level in percentage

In all endosphere and rhizosphere and control soil samples, the dominant bacterial phyla were *Pseudomonadota* (formerly *Proteobacteria*), *Bacteroidota* (formerly Bacteroidetes), and *Actinomycetota* (formerly *Actinobacteria)* (Fig. 1A, Additional file 1). *Pseudomonadota* was the most abundant phylum, comprising 35% of the bacterial community in soil and up to 94% in the rhizosphere during the flowering stage of the Tessema cultivar. *Bacteroidota* accounted for less than 1% of the rhizosphere during flowering (Tessema) and up to 37% in soil samples. *Actinomycetota* exhibited relative abundance ranging from 3% in the rhizosphere during flowering in Tadesse to 35% in the endosphere during flowering (Tadesse). However, *Pseudomonadota* was more abundant in the root compartments (including the endosphere and rhizosphere) than in the soil, whereas *Bacteroidota* showed the opposite trend. There were also notable differences in the relative abundance of these dominant phyla across root compartments and Plant developmental stages. The phylum *Actinomycetota* was more abundant in the endosphere (21%) than in the rhizosphere (9%). Similarly, *Bacteroidota* phyla showed higher relative abundance at the seedling stage (13%) than at the flowering stage (4%). Additionally, there was a clear shift in the rhizosphere microbiota composition between the seedling and flowering stages. At the flowering stage, *Pseudomonadota* was the sole dominant phylum, accounting for 84% of the bacterial community, whereas its relative abundance decreased to 60% at the seedling stage. Notable compositional differences existed between the cultivars (Fig. 1A). For example, during the flowering stage, the rhizosphere of the five domesticated cultivars showed a significantly higher abundance of *Pseudomonadota* than the wild type (Fig. 1A), contributing nearly 94% to the bacterial community composition compared to 49% in the wild type. Compositional shifts were also observed at lower taxonomic levels (genus), as shown in Fig. 1C. The *unclassified Rhodanobacteraceae* was among the top ten most abundant genera in all samples but was particularly enriched in the seedling samples. In contrast, the *Pseudomonas* genus exhibited low abundance during the seedling stage in both root compartments (Fig. 1C, Additional file 1), but showed a notable increase in the rhizosphere during the flowering stage. Similarly, the *unclassified Solirubrobacterales* were enhanced in abundance. Genotype-specific variations were also observed, such as high abundances of *unclassified Enterobacteriaceae* in the rhizosphere of cultivars Tessema, Tadesse, and Axum during the flowering stage.

Regarding the fungal community, *Ascomycota* and *Basidiomycota* were the most abundant fungal phyla in all samples, followed by *Mucoromycota* (Fig. 1B, Additional file 1). *Mortierellomycota* were also detected across all cultivars, Plant developmental stages and root compartments (Fig. 1B, Additional file 1). At the genus level, *Pseudogymnoascus*, *Candida* and *Chrysosporium* genera were dominant in the soil samples (Fig. 1D, Additional file 1). Rhizosphere samples were dominated by *Pseudogymnoascus*, *Apiotrichum*, *Candida* and *Umbelopsis* at both plant developmental stages (Fig. 1D). In contrast, the endosphere samples had a distinct set of dominant genera that included *Chrysosporium*, *Meliniomyces*, *Conlarium*, and *unclassified Helotiales* (Fig. 1D).

### Similarities and differences between cultivars in different plant developmental stages and root compartments

**Fig. 2:**
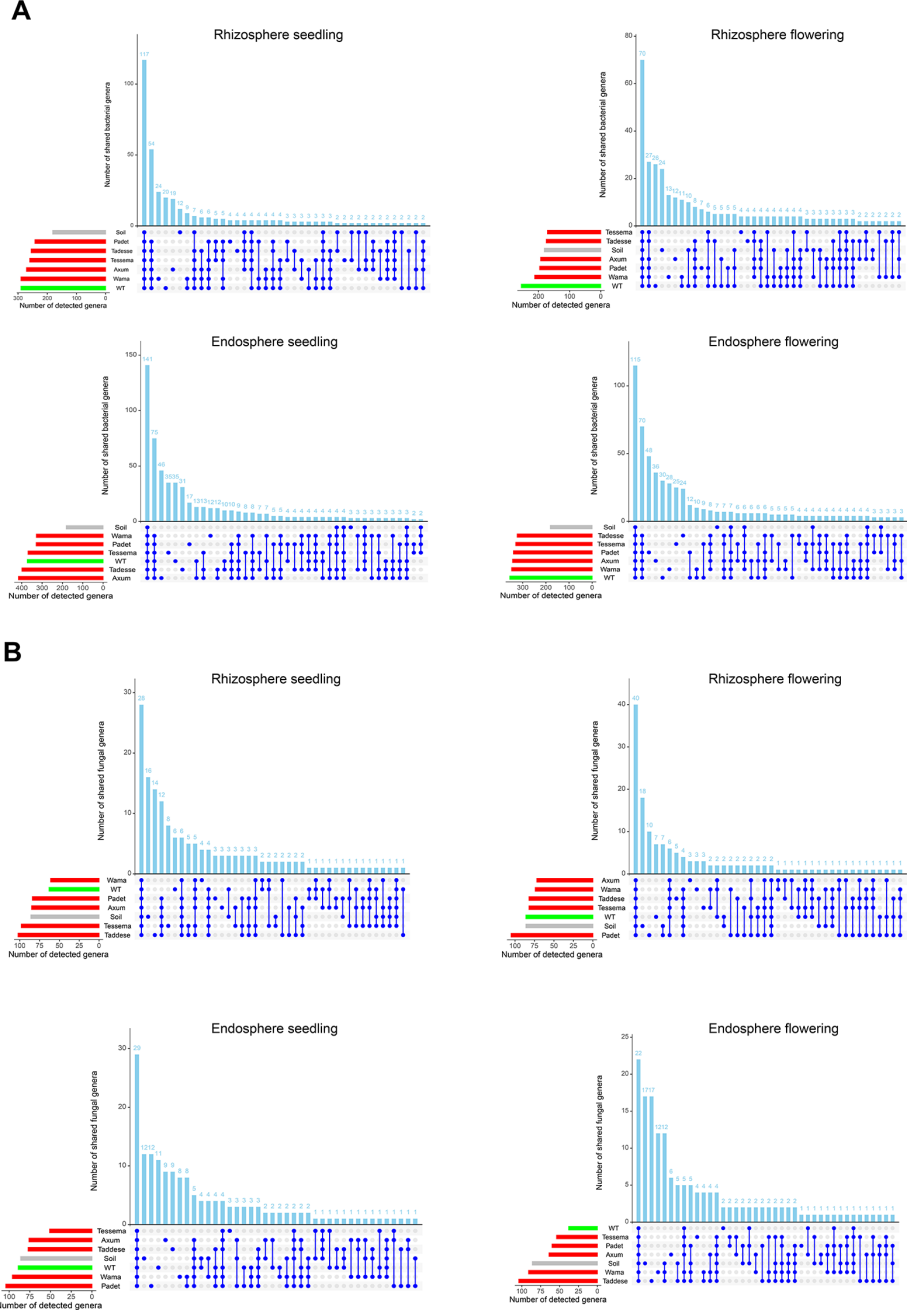
UpSet plot showing the results of core microbiome analyses of the bacterial genera (**A**) and fungal genera (**B**) present in the endosphere and rhizosphere of wild-type plants (WT; Africana), five different cultivars (Wama, Tessema, Tadesse, Padet, and Axum), and control soil (soil without plantation) during the seedling and flowering stages. The bars in the lower left corner and the columns at the top indicate the number of detected and shared genera. Connected dots represent genera shared among sample types (control, wild type, and cultivars), while single dots represent species unique to a specific sample type

A genus-level core microbiome analysis was conducted to identify shared and unique microbial genera. During the seedling samples, 117 (23.45%) bacterial genera were shared across all cultivars (including the wild type) and soil samples in the rhizosphere. Moreover, 54 genera were shared among the cultivars and the wild type but were not detected in the soil. The cultivar Wama had the highest number of 24 unique bacterial genera at the seedling stage (Fig. 2A). During the flowering stage, a shift in the core rhizosphere microbiome was observed, with the wild type exhibiting 26 unique bacterial genera. Only four genera were shared among all domesticated cultivars but were absent in the wild type and control soil. Furthermore, 70 genera were found in all samples, while 27 genera were shared between the soil and the cultivars (Fig. 2A).

In the endosphere during the seedling stage, 141 bacterial genera were shared among all the samples, including the soil (Fig. 2A). Seventy-five genera were detected among the cultivars but were absent in the control soil, while five genera were shared exclusively among the domesticated cultivars (Fig. 2A). During the flowering stage in the endosphere, 115 bacterial species were shared across all cultivars, including the wild type and soil, and 70 species were common to all cultivars but absent in the soil (Fig. 2A.). Overall, the endosphere bacterial communities had more shared genera with the soil than with the rhizosphere regardless of plant developmental stages.

In comparison, fewer fungal genera were shared among the samples than bacterial genera (Fig. 2B). In the rhizosphere during the seedling stage, 28 fungal genera were common across all samples (Fig. 2B), while the soil had 16 unique genera that were absent in the plants. Only five genera were shared among the plants. During the flowering stage, 40 fungal genera were shared across all samples, and six genera were detected in all the cultivars except for the soil (Fig. 2B).

In the endosphere during the seedling stage, the soil sample and Padet cultivar had more unique fungal genera (12) than the other cultivars. Twenty-nine genera were common across all sample types, including the control soil samples, while four genera were shared exclusively among the plants (Fig. 2B). In the flowering stage, the soil and Tadesse cultivar exhibited the highest number of unique genera (17) compared to the other cultivars (Fig. 2B).

### Core Microbiome

Further, core microbiome (i.e., a group of microbial taxa that are consistently present across all samples) analysis revealed that a significant proportion of microbiota in the root compartments of different cultivars is shared with the soil (Table 1). However, some microbial genera are unique to the cultivars and are absent in the soil. Across all cultivars, 62 bacterial genera (approximately 8%) were shared with soil microbiota (Table 1). The proportion of bacterial genera shared with the soil varied among individual cultivars, ranging from 17.2% (the lowest) in Tessema to 22.7% (the highest) in the wild type. Only nine bacterial genera were commonly present across all the cultivars, independent of soil bacterial communities. Furthermore, 20 fungal genera were shared between all cultivars and the soil, while only one fungal genus was commonly detected in all cultivars independent of the soil. Among the cultivars, the Tadesse exhibited a higher number of shared genera (39) than others (Table 1).

### Microbial diversity comparisons between cultivars and plant developmental stages

**Fig. 3:**
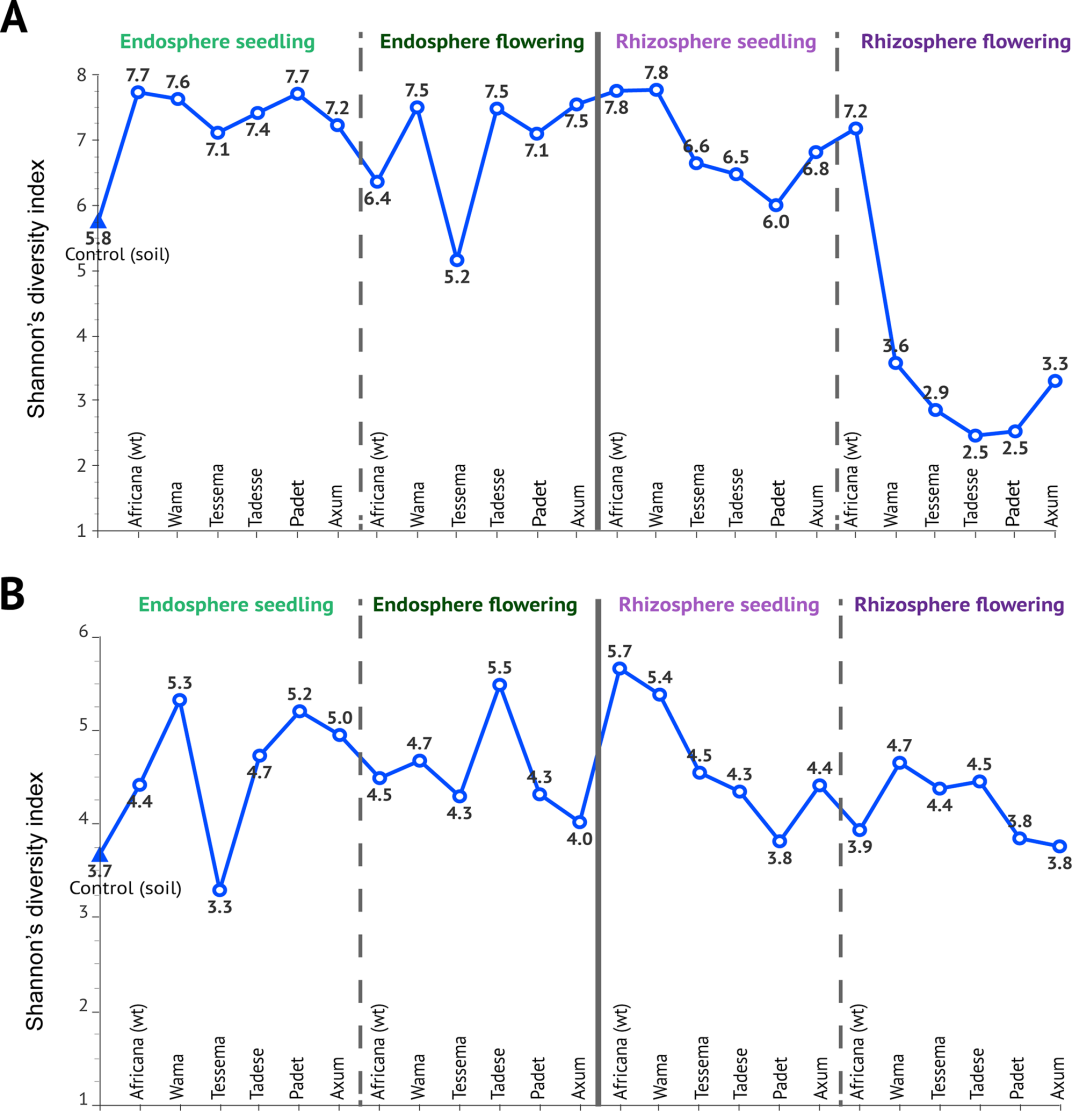
Changes in Alpha Diversity of Bacteriota and Mycobiota. This figure shows the alpha diversity (Shannon index on the Y axis) of bacteriota (**A**) and mycobiota (**B**), estimated using Shannon’s diversity index. The alpha diversity in the rhizosphere was clearly lower than in the endosphere during both the seedling (*p* = 0.049) and flowering stages (*p* < 0.001). Moreover, the alpha diversity of the rhizosphere declined significantly during the flowering stage (*p* < 0.001). No significant changes were observed in the endosphere or among with mycobiota (*p* = 0.186). The data are represented using median values for each sample type, including control (soil without plantation), wild type (Africana), and five domesticated cultivars (Wama, Tessema, Tadesse, Padet, and Axum). All *p*-values were adjusted for multiple comparisons

The endosphere bacteriota had similar levels of alpha diversity across all cultivars and the wild type during both the seedling and flowering stages Fig. 3A and Additional file 2. However, cv. Tessema had slightly lower alpha diversity in the endosphere during the flowering stage (Fig. 3A). The Padet cultivar had the lowest alpha diversity in the rhizosphere. Overall, the wild type maintained a high level of alpha diversity in the rhizosphere even during the flowering stage, when the other cultivars had significantly less diversity. However, there were no significant differences between cultivars concerning the beta diversity of the bacterial and fungal communities for any root compartment or developmental stage.

The alpha diversity of the rhizosphere and endosphere samples from the finger millet cultivars differed markedly from that in the control soil sample, as shown in Fig. 3A. In addition, there were significant differences in alpha diversity metrics (Shannon’s diversity Index) of the bacterial communities across different plant developmental stages and root compartments (Kruskal-Wallis test, followed by a post-hoc Mann–Whitney U test) Specifically, the rhizosphere exhibited lower bacterial alpha diversity during the flowering stage, showing significantly lower values than the endosphere at the same stage (*p* = 0.0006) than the endosphere. However, the bacterial alpha diversity of the rhizosphere in the seedling stage was higher and comparable to that of the endosphere (Fig. 3A). Remarkably, the wild-type finger millet maintained a higher alpha diversity than the cultivars in both the seedling and the flowering stages. In particular, the root endosphere consistently displayed higher levels of alpha diversity than any cultivar other than cv. Tessema irrespective of plant developmental stages (Fig. 3A).

In contrast, fungal alpha diversity was relatively consistent across both plant developmental stages and plant root compartments, with only a few cultivars showing variations (Fig. 3B). No significant differences were detected between the groups (Kruskal-Wallis test, followed by post-hoc Mann–Whitney U test, *p* = 0.186). However, the fungal alpha diversity in the rhizosphere during the flowering stage was slightly lower than in the bulk soil and the endosphere. In contrast to the bacterial diversity patterns, the fungal alpha diversity of the wild-type finger millet plants fluctuated throughout the study (Fig. 3B). Moreover, the drastic decline in alpha diversity seen in the bacterial community of the rhizosphere during the flowering stage was not seen in the corresponding mycobiota (Fig. 3B).

**Fig. 4:**
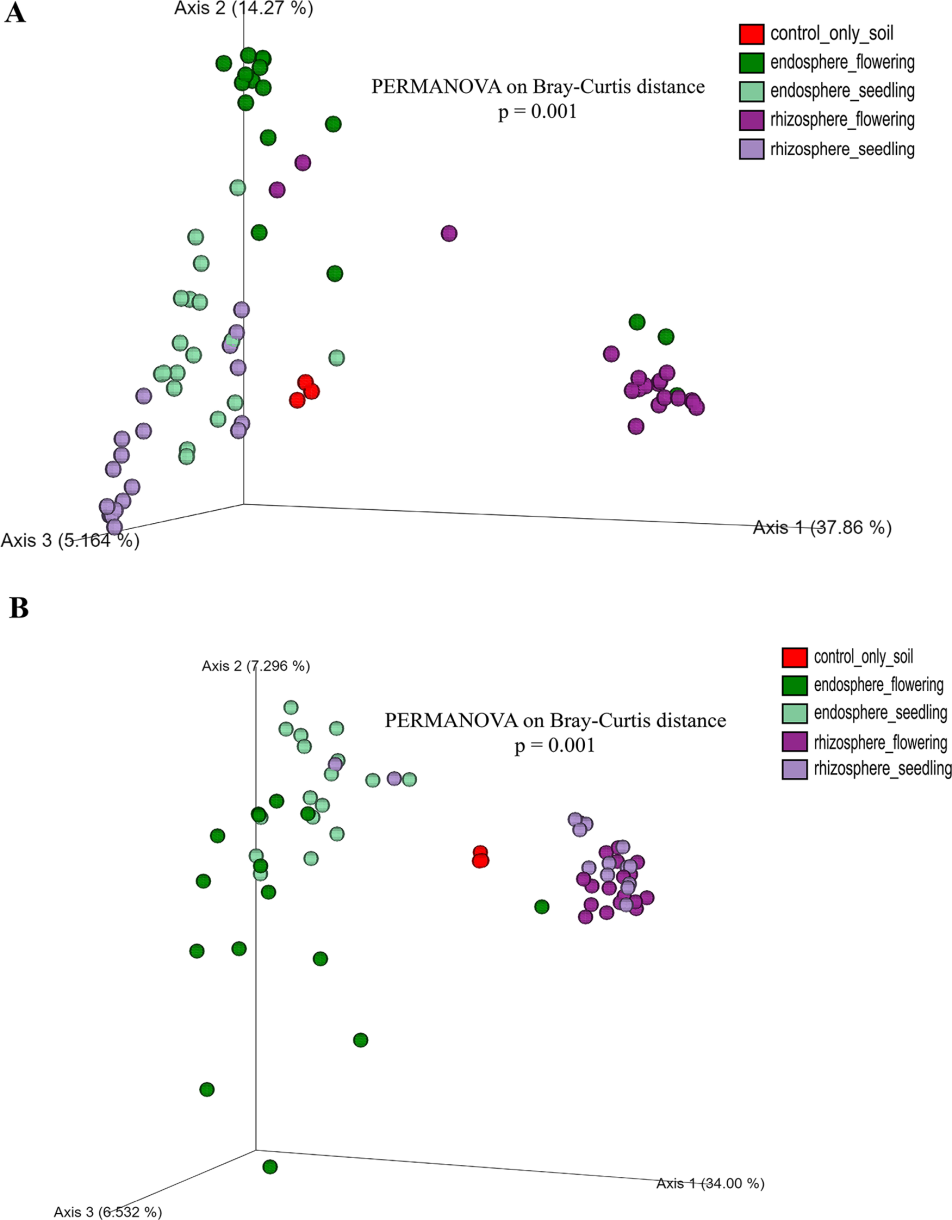
Differences in Beta Diversity of Bacteriota and Mycobiota. This figure shows the beta diversity of bacteriota (**A**) and mycobiota (**B**) in two plant compartments and plant developmental stages. Significant differences were found in all pairwise comparisons (*p* < 0.05; *p* values were corrected for multiple comparisons)

A beta diversity analysis based on the Bray‒Curtis distance matrix revealed significant differences between the rhizosphere and endosphere compartments at different stages of plant development (PERMANOVA, *p* = 0.001, Fig. 4A). Notably, the clustering pattern of the soil samples differed from that of the wild type and the cultivars. Furthermore, the microbial communities associated with the flowering stage had different clustering characteristics to the seedling-stage plants (Fig. 4A). A beta diversity analysis of the fungal community also revealed significant differences (PERMANOVA, p-value = 0.001) between samples in the rhizosphere (Fig. 4B). As previously observed for the bacterial communities, the pattern of the fungal communities in the rhizosphere differed from those in the endosphere (Fig. 4B).

## Discussion

### Influence of plant developmental stages and domestication on microbial composition and diversity in the rhizosphere and endosphere communities

The interaction between soil microbiomes and plant-root-associated microbial communities is vital for understanding both ecological and agricultural systems. Several factors influence this interaction, such as soil type, physiochemical properties, plant species, developmental stage, root exudates, and domestication processes [10, 15, 19–21, 30, 31]. These factors collectively shape the microbial communities associated with plant roots, affecting their composition, diversity, and plant health and growth. This study aimed to determine how the plant developmental stages (seedling and flowering) and domestication influence the microbial diversity within root compartments (endosphere and rhizosphere) of different finger millet cultivars (Wild type, Wama, Axum, Padet, Tessemma, Tadesse) and how these variations affect the community structure across the seedling and flowering stages.

### Microbial composition and community dynamics

Our study revealed distinct microbial community patterns between the rhizosphere and endosphere, influenced by plant developmental stages and domestication. These differences in microbial composition were apparent in both the bacteriota and the mycobiota (Fig. 1). The rhizosphere, enriched with nutrient-rich root exudates, was predominantly dominated by bacterial phyla such as P*seudomonadota*, *Actinomycetota*, and *Bacteroidota*. Among fungal taxa *Ascomycota* and *Basidiomycota* were predominant ones in both root compartments. At a lower taxonomic level, the bacterial genus *Pseudomonas* was more abundant in the rhizosphere during the flowering stage and, to a lesser degree, in the endosphere. Due to the wide spectrum of functional diversity associated with this genus [32, 33], which includes pathogens to plant-beneficial microbes, its increased abundance may reflect a complex ecological role. This may involve microbial competition, alteration of plant defense responses, or contributions to nutrient uptake and stress tolerance during this critical stage of development.

Meanwhile, the fungal genus Pseudogymnoascus showed a higher relative abundance in the rhizosphere, indicating specific microbial adaptations and shifts in community structure in response to root compartments. These findings support the idea that plant developmental stages and root compartments influence the composition of the finger millet root microbiome.

### Impact of developmental stages on microbiome composition

Our findings support previous research showing significant changes in plant-associated microbiomes across plant developmental stages [21, 34]. During the seedling stage, the rhizosphere’s bacterial alpha diversity (Shannon Index) was higher across all cultivars. However, there was a decrease in bacterial alpha diversity during the flowering stage, especially in domesticated cultivars. This may be due to the nutrient-rich exudates released during early growth, which can recruit diverse microbial populations that support rapid growth and defense against early pathogen attacks [21]. We cannot rule out the possibility that this reduction may also result from increased selective pressure due to physiological changes in plants, shifts in soil nutrient availability, microbial succession over time, or alterations in environmental interactions.

The decline in diversity also reflects the transition from seedling (vegetative) to flowering (reproductive); growth appears to coincide with a shift from a generalist to a more specialized microbial community that favors specific microbial taxa beneficial for reproductive growth. For example, during the flowering stage, the rhizosphere was enriched with *Pseudomonas*, a genus known for its diverse roles, including pathogens and beneficial plant microbes, such as pathogen suppression and stress management [35, 36]. While species-level resolution was not possible in this study, future work identifying key taxa more precisely would provide valuable insights into the functional roles of these microbes. Overall, our findings reveal dynamic shifts in the rhizosphere microbiome of finger millet across developmental stages, which may reflect interactions between plant developmental processes and microbial community dynamics.

Interestingly, the endosphere microbiome showed better stability across developmental stages than the rhizosphere, suggesting that plants maintain a core set of beneficial microbes internally. This contrasts with the rhizosphere, where microbial communities are more dynamic and influenced by changing exudate composition [9, 34]. Furthermore, fungal alpha diversity remained relatively consistent across plant developmental stages and root compartments, with only variations among cultivars (Fig. 3B). These differences were not statistically significant (Kruskal–Wallis test, followed by post-hoc Mann–Whitney U test, *p* = 0.186), suggesting that was not strongly influenced by these factors had a minimum influence on the fungal diversity under the given experimental conditions.

### Domestication and microbiome assembly

Domestication has been known to influence plant-associated microbiome composition and diversity; previous studies show that domestication often reduces microbial diversity due to selective breeding for traits like increased yield, disease resistance, or stress tolerance [20, 23]. This reduction in diversity may occur from domesticated plants that tend to produce a narrower range of root exudates or have altered immune responses that affect microbial recruitment [23]. Our study supports this observation, as domesticated cultivars (Wama, Axum, Padet, Tessemma, Tadesse) generally showed reduced microbial diversity within the endosphere. This reduction was more pronounced when compared to the wild type, especially during the flowering stage. This reduction suggests that domestication has led to the loss of specific (beneficial) microbial assemblages that help with nutrient uptake, improve plant resilience to stress, and protect against harmful pathogens over time due to selective pressure. As plants mature, these pressures may narrow the range of microbial communities in specific root compartments, potentially making them more vulnerable to diseases, reducing their growth potential, and negatively impacting overall plant health. This could be linked to the production of fewer types of root exudates or altered immune responses that affect microbial recruitment [37]. This reduction in microbial diversity was particularly evident in the endosphere of domesticated cultivars, which aligns with studies showing that domestication can narrow the microbial community dynamics [10, 29].

Further, our study provides additional insights into how microbial communities shift over time and across developmental stages. We observed reduced microbial diversity in domesticated cultivars, particularly in the flowering stages, where an abundance of specific genera was enriched. However, due to the lack of species-level resolution, it remains uncertain whether this enriched genus is beneficial, commensal or pathogenic. These trends mirror observations in other crops, where selective breeding has influenced the microbes associated with plant resilience against environmental conditions [22, 29, 38]. However, lacking more detailed genetic or phenotypic information about the individual cultivars, it remains challenging to establish the exact linkage between these microbial patterns and specific cultivar characteristics.

### Root compartment (including endosphere and rhizosphere) effects on microbial communities

The rhizosphere and endosphere harbor distinct microbial communities consistent with previous studies [16, 39, 40]. The rhizosphere, a nutrient-rich environment that attracts a variety of microorganisms, supports higher microbial diversity through root exudates [41]. In contrast, the endosphere is more selective, influenced by the plant’s immune system and the microbes’ ability to colonize root tissues [9, 12].

Our findings confirm these compartments’ specific microbial compositions; the rhizosphere was dominated by taxa such as *Pseudomonas* (bacteria) and *Pseudogymnoascus* (fungal) during flowering, which is involved in nutrient cycling, pathogen suppression and stress resistance [42, 43]. While, bacterial phyla *Actinomycetota* dominated the endosphere also, we observed selective enrichment of endophytic fungi such as *Chrysosporium*, *Phialocephala*, and *Meliniomyces*, which are known for promoting plant growth and enhancing stress tolerance [44–46]. Moreover, the endosphere shares more bacterial genera with soil than the rhizosphere, implying that the endosphere community may reflect a more stable and selectively filtered subset of soil microbiota. Soil is a primary reservoir for both the rhizosphere and endosphere; however, the recruitment process differs between root compartments. While the rhizosphere is shaped by environmental factors such as root exudates that selectively enrich specific taxa capable of utilizing particular compounds [19], the endosphere is influenced by more stringent filtering mechanisms, allowing only a limited number of physiologically adapted microbes to colonize internal root tissues [17].

Interestingly, while previous studies have generally reported higher microbial diversity in the rhizosphere compared to the endosphere [41, 47], our results revealed a reduction in bacterial alpha diversity during the flowering stage (Fig. 3A). This discrepancy could be associated with plant developmental stage transitions and selective processes in plant compartments, such as changes in nutrient availability, symbiotic interactions, and stress responses. The reduced microbial diversity in the rhizosphere during flowering was accompanied by the strong enrichment of a single genus, suggesting intense selection pressure by the host plant. These findings suggest that variations in microbial community composition between the rhizosphere and endosphere are driven by differences in nutrient availability and selective pressures, promoting the recruitment of specific microbes suited to each environment.

### Successional shifts and core microbiomes

Our study also highlights the successional shifts in microbial communities across plant developmental stages. A beta-diversity analysis revealed significant differences in microbial composition between the seedling and flowering stages, suggesting that the plant developmental stage significantly influences microbial diversity more than the plant’s genotype [34]. These successional shifts indicate that plants alter their microbial assemblages as they mature, possibly to fulfill their physiological needs and exudate profiles.

While the rhizosphere microbiome was highly dynamic, the endosphere microbiome remained more stable across plant developmental stages. This supports the hypothesis that plants exert selective pressure on endophytic microbes, maintaining a core microbiome that promotes plant health throughout development. Moreover, our study also showed that the endosphere microbiome shared more bacterial genera across different cultivars than the rhizosphere, suggesting its central role in plant development and stress resilience. This stability could be due to the protective environment within the endosphere, which reduces exposure to external environmental fluctuations and allows a core set of microbial communities to thrive across different cultivars. Furthermore, plants selectively allow only microbes that can evade their immune defenses to colonize the endosphere, leading to a consistent microbial community throughout various plant developmental stages. Endophytic communities might initially be recruited from the surrounding soil and rhizosphere, and once they enter the endosphere, they may become a more consistent microbial community. As plants grow through various stages, the endosphere microbiome remains stable, while the rhizosphere microbiome responds dynamically to changes in root exudates.

Our results showed lower overall fungal diversity than for bacteria, which may be partly due to limitations in assigning many fungal sequences to known taxa. We observed cultivar-specific recruitment of fungal species, particularly in the rhizosphere, where genera like *Pseudogymnoascus* were enriched. The limited taxonomic resolution for fungi restricts detailed analysis of fungal dynamics, making it more challenging to draw clear conclusions than for bacterial communities.

Our study demonstrates that both plant developmental stages and domestication significantly influence the microbial diversity and composition of root-associated communities in finger millet. However, the stronger influence on microbial diversity and composition was influenced by plant developmental stage than other factors such as cultivar genotype and root compartment. The findings suggest that plants actively shape their root microbiomes in response to changing environmental conditions and growth requirements. Domestication has reduced the overall diversity of root-associated microbiomes while selectively enriching beneficial microbial taxa.

## Conclusion

Our study confirms that plant developmental stages and domestication influence root-associated microbial communities and provides new insights into how these communities change over time. Unlike previous research that focused on one-point comparisons, this study demonstrates how microbial diversity and composition shift dynamically over plant developmental stages, especially during the flowering stage, when plants transition from the vegetative stage to the reproductive stage. We found that domestication reduces microbial diversity, particularly in the flowering stage. Domesticated plants selectively recruit microbes that enhance stress tolerance and nutrient uptake. This contrasts with some studies that found no significant loss in diversity during domestication, suggesting that these effects are more pronounced in later plant developmental stages.

## Root

 exudates drive changes in microbial communities that plants modify according to their developmental stage and environment. Future research using metagenomics and metabolomics could identify key microbial taxa and functional pathways that mediate plant-microbe interactions across different developmental stages. These insights have practical implications for breeding programs focused on enhancing beneficial plant-microbe interactions to support crop resilience, reduce chemical inputs, and promote sustainable crop productivity. Overall, our findings highlight the influence of both cultivar type and developmental stage in shaping root-associated microbiomes, offering a deeper understanding of how domestication dynamically influences plant-microbe interactions.

## Materials and methods

### Finger millet sources and growth conditions

The African finger millet plant species used in this study include the wild type *Eleusine coracana* subsp. *Africana* (referred to as Africana in the following text and figures) and the domesticated *Eleusine coracana* subsp. *Coracana* (represented by the cultivars Axum, Wama, Padet, Tesema, and Tadesse). These were obtained from the Melkassa Research Center, Ethiopian Institute of Agricultural Research (EIAR), Ethiopia. Detailed information on these varieties is given in Table 2. Finger millet seeds were subjected to surface sterilization using a standardized disinfection protocol before sowing to eliminate microbes not present within the endophytic seed-transmittable microbiome. Initially, the seeds were rinsed twice in a 0.1% (v/v) Tween 20 solution for 5 min under continuous agitation to remove surface contaminants. This was followed by immersion in a 15% (v/v) commercial sodium hypochlorite (bleach) solution for 10 min with constant agitation to ensure thorough sterilization. The seeds were subsequently washed seven times with sterile distilled deionized water to eliminate any residual disinfectants. Plants were cultivated in a commercially available soil mix, Emmaljunga Exclusive Bloom and Planting Soil, produced by Emmaljunga Torvmull AB, designed for indoor plant cultivation. The soil composition included 50% light peat, 33% dark peat, 7% sand, 5% clay with silicon, and 5% play balls (2–4 mm), with a fine to medium texture. The soil had an approximate pH of 5.5–6.5 and an electrical conductivity of 2.0–4.0 mS/cm. Additionally, per cubic meter of soil, 5.5 kg of limestone flour, 1.5 kg of NPK fertilizer (11-5-18) with micronutrients, 200 g of extra micronutrients, and 100 g of long-acting iron were incorporated to enhance nutrient availability. Plants were cultivated without fertilizer in 7.5-liter pots, with three plants per pot, under controlled conditions in the SLU Biotron chamber in Alnarp, Sweden. The growth environment featured day and night temperatures of 30/27°C, a 12-hour light/dark cycle, and a light intensity of 350 µmol m^− 2^ s^− 1^. Three biological replicates of plants from each cultivar and treatment were grown.

**Table 1:** Core microbiota present in both root compartments from seedling to flowering stage. Data presented were number of shared bacterial or fungal genera and proportion of the detected genera (%)

	All cultivars		Individual cultivars												
	Present in all (Soil + All cultivars)	**Plant specific (Absent in soil)**	Wild type		**Wama**		Tessema		Tadesse		Padet		Axum		
Shared with soil bacteria	62 (7.8%)	NA	118 (22.7%)		99 (20.8%)		86 (17.2%)		93 (19.0%)		100 (21.4%)		101 (19.6%)		
Independent of soil bacteria	NA	9 (1.1%)	55 (10.6%)		39 (8.3%)		32 (6.5%)		32 (6.6%)		39 (8.6%)		35 (6.9%)		
Shared with soil fungi	20 (8.2%)	NA	21 (14%)		32 (20.4%)		28 (18.4%)		39 (22.8%)		34 (21.5%)		28 (17.8%)		
Independent of soil fungi	NA	1 (0.4%)	3 (2.3%)		5 (3.6%)		1 (0.8%)		5 (3.2%)		4 (2.8%)		2 (1.4%)		

NA: not applicable

**Table 2:** Details of the cultivars and their characteristics

Name of the cultivars	Year of release	Maintainer	Characteristics
Tadesse	1999	MARC	Grain yield
Wama	2007	BARC	Resistance for head blast disease
Axum	2016	MARC	Resistance to blast disease
Tessema	2014	MARC	Grain Yield
Padet	1998	MARC	Tolerant to blast disease

### Sample collection

#### Collection of root endosphere and rhizosphere samples

Samples from each plant were collected at two different Plant developmental stages for metagenomic analysis targeting the 16 S rRNA gene and internal transcribed spacer (ITS) region. Rhizosphere and root endosphere samples were collected at two plant developmental stages. The first sampling was done during the vegetative (seedling) stage, i.e., eight weeks after sowing. The second sampling was done during the flowering stage (i.e., five months after sowing), once the plants had shifted from the vegetative to the reproductive stage. The experiment was run from March to July 2022. For each plant’s developmental stage, three biological replicates were collected by excavating one plant from each of three separate pots and the roots were collected. However, the bulk soil sample was not collected as part of this study. In total, 72 samples (i.e., 6 genotypes × 2 stages × 2 compartments × 3 replicates = 72). The non-rhizosphere (loosely attached) soil was separated from the roots by shaking. The collected root samples were placed in 50 mL tubes containing 35 mL of sterile phosphate buffer solution (6.33 g/L NaH_2_PO_4_, 8.5 g/L Na_2_HPO_4_ anhydrous, 200 µL/L Tween 20, pH 6.5). The tubes were vigorously shaken for 2 min to release rhizosphere soil from the roots, after which the roots were removed, blotted on paper towels, and placed in new, labeled 50 mL tubes for endosphere sampling. The rhizosphere soil tubes, and excised root samples were placed on dry ice, transported to the laboratory and stored at -80 °C before for further processing. Control samples (triplicates) were obtained by collecting 250 g of soil from each pot before sowing for DNA extraction.

### Sample preparation

Root samples were surface sterilized by washing roots with 50% bleach and 0.01% Tween 20 to remove physical impurities and vigorously shaken for 30 s. The root segments were then rinsed with 35 mL of 70% ethanol for one minute, followed by three rinses with sterile water, and dried on clean paper towels, as mentioned earlier [48]. The effectiveness of the sterilization was confirmed by culturing aliquots from the final wash on potato dextrose agar and nutrient agar (Sigma-Aldrich). Rhizosphere samples were filtered through a sterile 100 μm mesh filter (Fisher Scientific, Waltham, MA, USA) to remove larger plant debris into a sterile 50 mL tube and pelleted at 3000× *g* for 10 min at room temperature and then resuspended in phosphate buffer. The rhizosphere was pelleted again by centrifuging the tubes at 15,000× *g* at 4 °C for 10 min. Finally, the rhizosphere pellet was stored at -20 °C until DNA extraction.

### DNA extraction and next-generation sequencing (NGS)

DNA was extracted from 36 endosphere and 36 rhizosphere soil samples using the DNeasy Power Soil Pro Kit (Qiagen, CA, USA) following the manufacturer’s instructions. Root endosphere samples were briefly ground to powder using a Mixer Mill MM 400 Shaker (Retsch, UNSPSC, UK) for 5 min with glass beads (5 mm diameter) in a grinding jar. Lysis buffer C1 was added to the pelleted rhizosphere soil and ground root samples, which were homogenized with FastPrep-24 (MP Biomedicals, USA). The quality and quantity of the extracted DNA were assessed using a Nanodrop (Nanodrop 8000 Thermo Fisher Scientific, USA). For bacteria, the V5-V6 regions of the bacterial 16 S rRNA gene were amplified using the primer pair 799 F (AACMGGATTAGATACCCKG) and 1115R (AGGGTTGCGCTCGTTG). For fungi, the internal transcribed spacer 2 (ITS2) region was targeted by the primer pair fITS7 (GTGARTCATCGAATCTTTG) and ITS4 (TCCTCCGCTTATTGATATGC) [49, 50]. For each sample, PCR reactions were performed using a 20 µL mixture containing 1x MyTaq buffer containing 1.5 units of MyTaq DNA polymerase (Bioline GmbH, Luckenwalde, Germany), 2 µl of BioStabII PCR Enhancer (Sigma‒Aldrich Co.), ~ 1–10 ng of template DNA, and 15 pmol of the appropriate forward and reverse primers. PCR was performed using the following program: predenaturation at 96 °C for 1 min, denaturation at 96 °C for 15 s, annealing at 55 °C for 30 s, and extension at 70 °C for 90 s, followed by 30–33 cycles of amplification for bacteria and 35–40 cycles for eukaryotic fungi. The DNA concentration of the amplicon was assessed by gel electrophoresis. The amplified PCR products were purified with Agencourt AMPure beads (Beckman Coulter, Brea, CA, United States) to remove primer dimers and other small mispriming products, and further purification was performed with MiniElute columns (QIAGEN GmbH, Hilden, Germany). The purified amplicon pool DNA (100 ng each) was used to construct Illumina libraries using the Ovation Rapid DR Multiplex System 1–96 (NuGEN Technologies, Inc., California, USA). After library construction, the Illumina libraries (Illumina, Inc., CA, USA) were combined and subjected to size selection by preparative gel electrophoresis. Sequencing was performed on an Illumina MiSeq platform at the LGC’s sequencing facility in Berlin (LGC Genomics GmbH, Germany).

### Bioinformatics and statistical analysis

Sequence data analysis was performed using the open-source bioinformatics tool QIIME 2 2024. 5 Distribution [51]. Briefly, adapter and primer sequences were trimmed off using the cutadapt plugin [52] and the trimmed sequences were processed with the dada2 plugin [53]. All identified amplicon sequence variants (ASVs) were analyzed further. To classify bacteria, a naïve Bayes classifier was trained using the V5-V6 region of the reference sequences from SILVA138.1 using the QIIME2 plugin feature classifier [54–56]. The fungal classification was performed using a pre-trained classifier on the UNITE reference database provided by the QIIME2 development team [57].

Alpha diversity was estimated using Shannon’s diversity index, beta diversity was calculated using the Bray‒Curtis distance matrix, and PERMANOVA was used for statistical testing. These analyses were performed using QIIME2 [51]. Core microbiome analysis was performed in RStudio (RStudio 2023.06.1 Build 524) using the UpSetR package [58]. P-values were adjusted for multiple comparisons using the Benjamini–Hochberg method with a statistical significance threshold of *P* < 0.05.

## Electronic supplementary material

Below is the link to the electronic supplementary material.

Supplementary Material 1: Additional file 1. Relative abundance of each bacterial and fungal phylum and genus from two root compartments (including the endosphere and rhizosphere) of six finger millet cultivars at two plant developmental stages

Supplementary Material 2: Additional file 2. Rarefaction curves of wilt types and domesticated cultivars during plant developmental stages and root compartments (including the endosphere and rhizosphere). The sampling depths were approximately 10,000 reads per sample

## Data Availability

All amplicon data used in this study were deposited into the sequence read archive (SRA) under BioProject accessionPRJNA1061570.
