# Supplementary figures and images for: Plant development influences dynamic shifts in the root compartment microbiomes of wild and domesticated finger millet cultivars
Source: BMC Microbiol. 2025 Apr 30;25:259. doi: 10.1186/s12866-025-03976-8 (PMC12042305; doi:10.1186/s12866-025-03976-8)

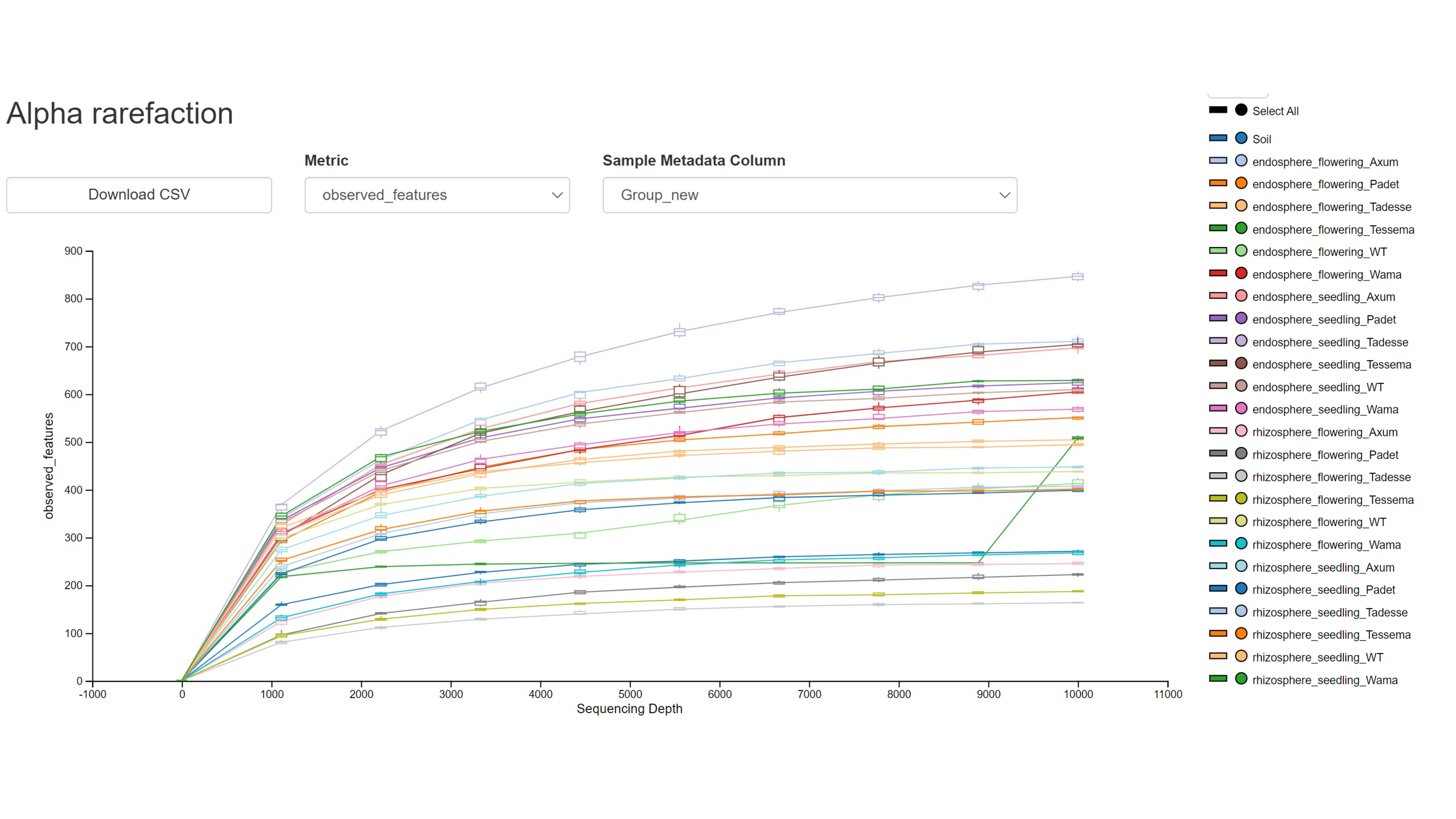

Supplement: Supplementary file 2 — Supplementary Material 2: Additional file 2. Rarefaction curves of wilt types and domesticated cultivars during plant developmental stages and root compartments (including the endosphere and rhizosphere). The sampling depths were approximately 10,000 reads per sample [file 12866_2025_3976_MOESM2_ESM.jpg]
